# Rap1b but not Rap1a in the forebrain is required for learned fear

**DOI:** 10.1186/s13578-020-00469-1

**Published:** 2020-09-11

**Authors:** Wen-Bing Chen, Han-Qing Pan, Ye He, Xue-Hui Wang, Wen-Hua Zhang, Bing-Xing Pan

**Affiliations:** 1 School of Life Sciences, Nanchang University, Nanchang, 330031 China; 2 Institute of Life Science, Nanchang University, Nanchang, 330031 China; 3 Center for Basic Medical Experiment, Nanchang University, Nanchang, 330031 China; 4 Department of Ophthalmology, The 2nd Affiliated Hospital of Nanchang University, Nanchang, 330006 China

**Keywords:** Rap1, Learned fear, Innate fear, Amygdala, mPFC

## Abstract

**Background:**

Fear is an adaptive response across species in the face of threatening cues. It can be either innate or learned through postnatal experience. We have previously shown that genetic deletion of both Rap1a and Rap1b, two isoforms of small GTPase Rap1 in forebrain, causes impairment in auditory fear conditioning. However, the specific roles of these two isoforms are not yet known.

**Results:**

In the present study, employing mice with forebrain-restricted deletion of Rap1a or Rap1b, we found that they are both dispensable for normal acquisition of fear learning. However, Rap1b but not Rap1a knockout (KO) mice displayed impairment in the retrieval of learned fear. Subsequently, we found that the expression of c-Fos, a marker of neuronal activity, is specifically decreased in prelimbic cortex (PL) of Rap1b KO mice after auditory fear conditioning, while remained unaltered in the amygdala and infralimbic cortex (IL). On the other hand, neither Rap1a nor Rap1b knockout altered the innate fear of mice in response to their predator odor, 2,5-Dihydro-2,4,5-Trimethylthiazoline (TMT).

**Conclusion:**

Thus, our results indicate that it is Rap1b but not Rap1a involved in the retrieval process of fear learning, and the learned but not innate fear requires Rap1 signaling in forebrain.

## Background

Fear is an adaptive response across species upon the emergence of threatening cues in environment and is critical for survival [1]. It can be either inherited (known as innate fear) or achieved through learning during postnatal life (learned fear) [2]. In the past decades, understanding how fearful response is formed in the face of threat has attracted wide attention, partly due to the increasing prevalence of fear-related neuropsychiatric disorders [1, 3]. Studies in both human being and animals have shown that innate and learned fear share much in the circuit mechanisms that mediate fear-related behaviors like “fight-or-flight” [1, 4]. However, the roles of specific molecules in these two forms of behaviors may vary dramatically. For example, stathmin and acid-sensing ion channel 1, two amygdala-enriched genes, were shown to be critical for both innate and learned fear [5, 6]. In contrast, zinc transporter 3, another amygdala-concentrated gene, was only required for learned but not innate fear [7]. Furthermore, BDNF in prelimbic cortex, a region critical for fear behavior, was also shown to be required selectively for learned fear [8].

Rap1, an important member of small GTPase Ras family, is ubiquitously expressed in various tissues and is involved in a variety of cellular processes including cell adhesion, proliferation and differentiation, migration and polarity [9–12]. In the central nervous system, Rap1 has been implicated in neuronal development [13, 14], synaptic plasticity and neuronal excitability [15]. Rap1 contains two isoforms, Rap1a and Rap1b. By generating mice of double knockout of Rap1a and Rap1b, we have shown that Rap1 is important for the appropriate expression of learned fear [16]. However, it remains unknown whether there is a specific role of Rap1a and Rap1b in learned fear. Besides, whether the other type of fear, the innate fear, requires Rap1 should also be determined.

To address these questions, we here generated mice with forebrain-specific knockout (KO) of Rap1a or Rap1b gene using the Cre-loxP system. We found that both Rap1a KO and Rap1b KO mice exhibited normal acquisition of fear learning. However, remarkably, deficits in fear retrieval were observed in Rap1b KO mice, while not in Rap1a KO mice, indicating an essential role of Rap1b in fear retrieval. Next, we examined the neuronal activity in the amygdala and medial prefrontal cortex (mPFC), two key brain regions in fear conditioning [17–21]. After fear memory retrieval, Rap1b KO mice exhibited reduced c-Fos expression in the prelimbic (PL) subregion of mPFC, without any changes in the amygdala and infralimbic (IL) subregion of mPFC, suggesting that Rap1b might underlie the increased PL neuronal activity to maintain the expression of learned fear. Furthermore, both Rap1a KO and Rap1b KO mice showed normal innate fear to predator odor. Overall, our findings reveal that learned but not innate fear requires Rap1, mainly Rap1b, in the forebrain.

## Results

### Characterization of mice with forebrain-restricted knockout of Rap1a or Rap1b

**Fig. 1:**
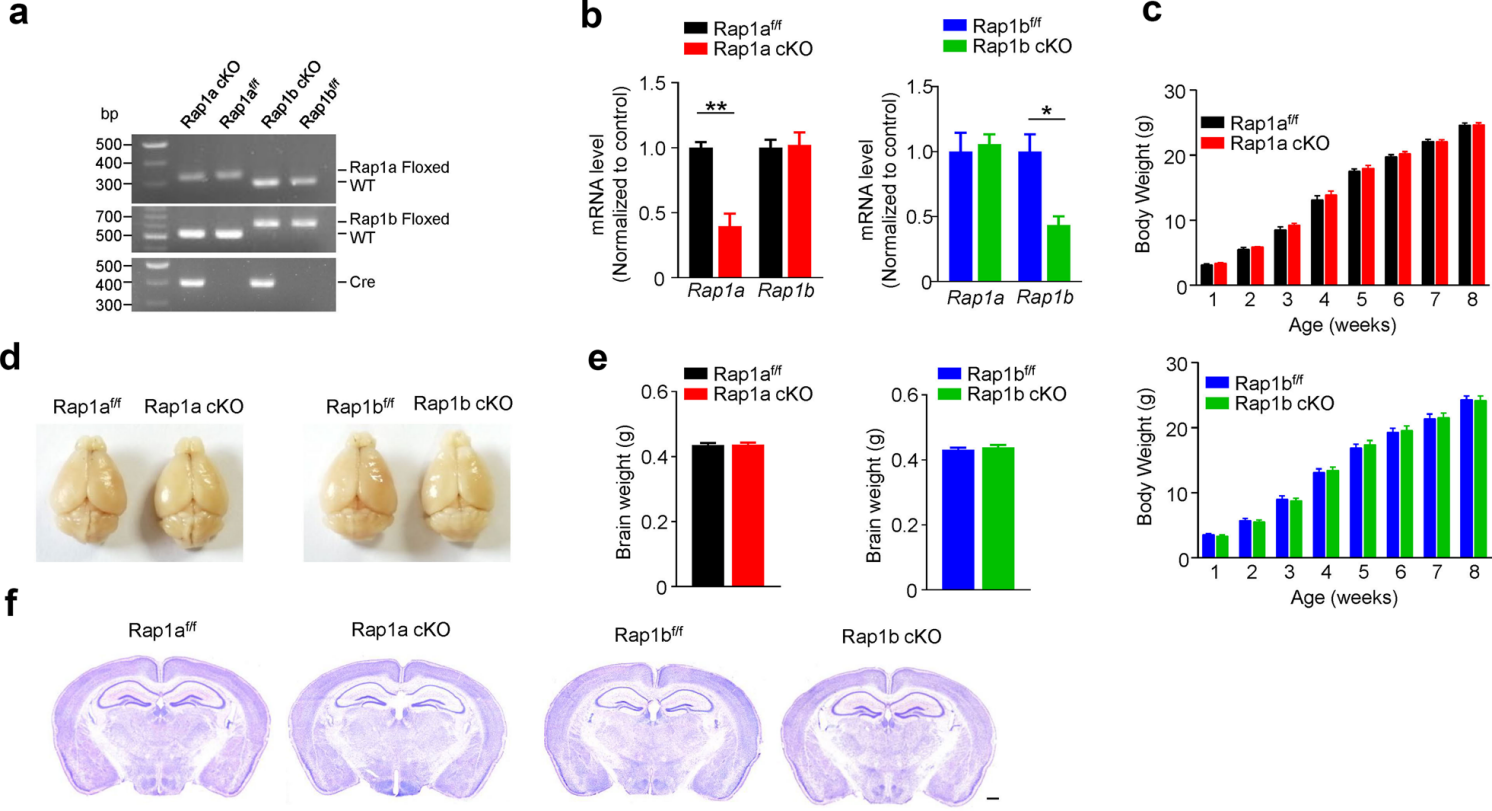
Generation of forebrain-specific Rap1a or Rap1b knockout mice. **a** Genomic DNA PCR genotyping of Rap1a^f/f^, Rap1b^f/f^, Rap1a cKO and Rap1b cKO mice. Arrows indicate bands expected for different genotypes. **b** Decreased mRNA levels of *Rap1a* without change in *Rap1b* in the cortex of Rap1a cKO mice (Left), decreased mRNA levels of *Rap1b* without change in *Rap1a* in the cortex of Rap1b cKO mice (Right). n = 3 mice for per genotype; Gapdh was used as loading controls. **c** Conditional deletion of either Rap1a or Rap1b in the forebrain had no effect on mouse body weight. n = 9 or 10 mice per group. Rap1a^f/f^ group, Rap1a cKO group and Rap1b cKO group (n = 14 mice); Rap1b^f/f^ group (n = 15 mice). **d** Images of the whole brain at P56. **e** Rap1a or Rap1b knockout had no effect on brain weight. n = 9 or 10 mice per group. **f** Rap1a or Rap1b knockout had no effect on gross morphology of brain. Brain were subjected to Nissel staining at P56. Scale bar, 500 μm. Data are presented as mean ± SEM; *p < 0.05, **p < 0.01

We have previously generated mice with double knockout of Rap1a and Rap1b from forebrain and found that these mice displayed moderate impairment in auditory fear conditioning [16]. To further clarify the specific role of these two isoforms in fear learning, we hereby selectively deleted Rap1a or Rap1b from forebrain by crossing Rap1a^f/f^ or Rap1b^f/f^ mice with the CaMKIIα-Cre mice, here after referred to as Rap1a cKO (Rap1a^f/f^; CaMKIIα-Cre) and Rap1b cKO (Rap1b^f/f^; CaMKIIα-Cre), respectively (Fig. 1a). Quantitative real-time PCR showed the rap1a mRNA in the cortical regions of Rap1a cKO mice was significantly reduced, while leaving rap1b mRNA unaffected, compared with Rap1a^f/f^ mice (unpaired student’s *t* test, For rap1a, Rap1a^f/f^ (1.00 ± 0.04) vs Rap1a cKO (0.40 ± 0.10); *p* = 0.005; For rap1b, Rap1a^f/f^ (1.00 ± 0.06) vs Rap1a cKO (1.02 ± 0.10); *p* = 0.870) (Fig. 1b). Vice versa, in Rap1b cKO mice, the expression of rap1b mRNA was reduced while rap1a was unaltered (unpaired student’s *t* test, For rap1a, Rap1b^f/f^ (1.00 ± 0.14) vs Rap1b cKO (1.06 ± 0.08); *p* = 0.753; For rap1b, Rap1b^f/f^ (1.00 ± 0.13) vs Rap1b cKO (0.44 ± 0.07); *p* = 0.019) (Fig. 1b). Compared with their WT littermates, the bodyweight of cKO mice showed a similar increase during development (*p* > 0.05 in all comparisons) (Fig. 1c). Moreover, at adult stage, cKO mice also showed normal brain size, weight and gross morphology when compared to their WT littermates (Fig. 1d–f), suggesting that neither Rap1 nor Rap1b cKO in forebrain affects the gross development of mice.

### Rap1b but not Rap1a is required for the retrieval of learned fear

**Fig. 2:**
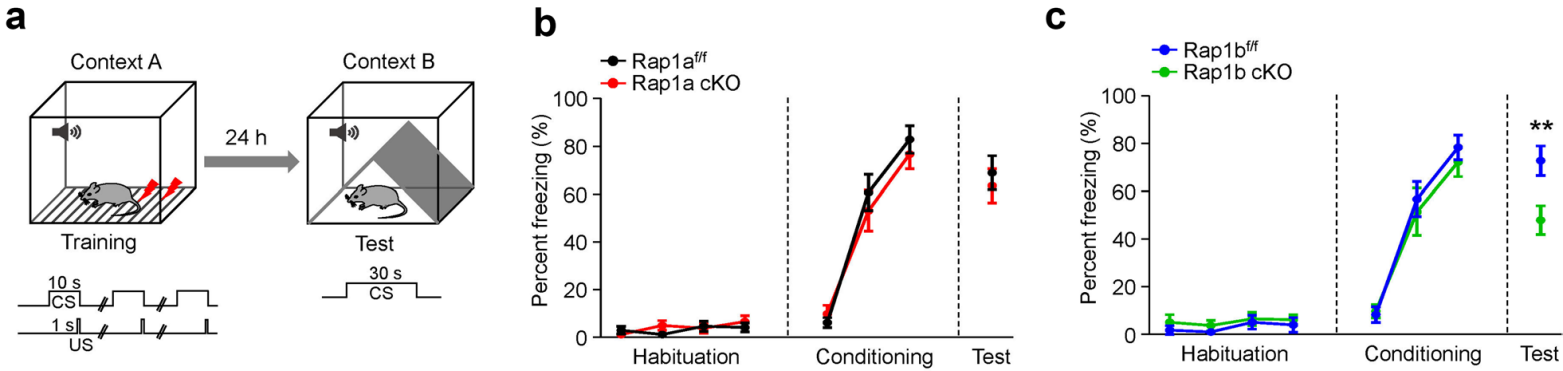
Fear memory deficits in Rap1b cKO but not in Rap1a cKO mice. **a** Schematic diagram of tone-fear conditioning in the present study. In 1st day, mice were subjected to 4 times CS without US for habituation and were subsequently 3 times CS-US paired for fear acquisition in context A. 24 h later, fear memory test was conducted by placing mice in context B. **b** No difference of freezing response during habituation, fear conditioning and test between Rap1a^f/f^ and Rap1a cKO mice. n = 14–15 mice per group. **c** Impaired fear memory expression in Rap1b cKO mice, compared with Rap1b^f/f^ mice. n = 12 mice per group. Data are presented as mean ± SEM; **p < 0.01

We next tested the specific roles of Rap1a or Rap1b in fear learning. We first used a training protocol comprised of 4 conditioned stimulus (CS) and 3 CS—unconditioned stimulus (US) pairings (Fig. 2a). In the training chamber, both Rap1a and Rap1b cKO mice displayed similar- and low-level of basal freezing during CS habituation (*Two-way* ANOVA, Rap1a^f/f^, n = 14, Rap1a cKO, n = 15, *F*_(1, 108)_ = 0.473; *p* = 0.493; Rap1b^f/f^, n = 12, Rap1b cKO, n = 12, *F*_(1, 88)_ = 1.893; *p* = 0.172), as well as similar increase of freezing when CS was presented (*Two-way* ANOVA, Rap1a, *F*_(1, 81)_ = 464, *p* = 0.498; Rap1b, *F*_(1, 66)_ = 0.422, *p* = 0.518) (Fig. 2a, b), compared with their control littermates. This result indicates that Rap1a or Rap1b cKO in forebrain did not affect fear acquisition. 24 h later, the mice were placed in a different testing context with the presence of a continuous 30-s CS. Compared to their control littermates, the Rap1a cKO mice exhibited similar freezing level (unpaired student’s *t* test, Rap1a^f/f^, n = 14, Rap1a cKO, n = 15; *p* = 0.594) (Fig. 2c). By contrast, Rap1b cKO mice displayed less freezing time (unpaired student’s *t* test, Rap1b^f/f^, n = 12, Rap1b cKO, n = 12; *p* = 0.009). These results suggest that Rap1b, but not Rap1a, mediates the retrieval of learned fear.

### Aberrant neuronal activation in the PL of Rap1b cKO mice

**Fig. 3:**
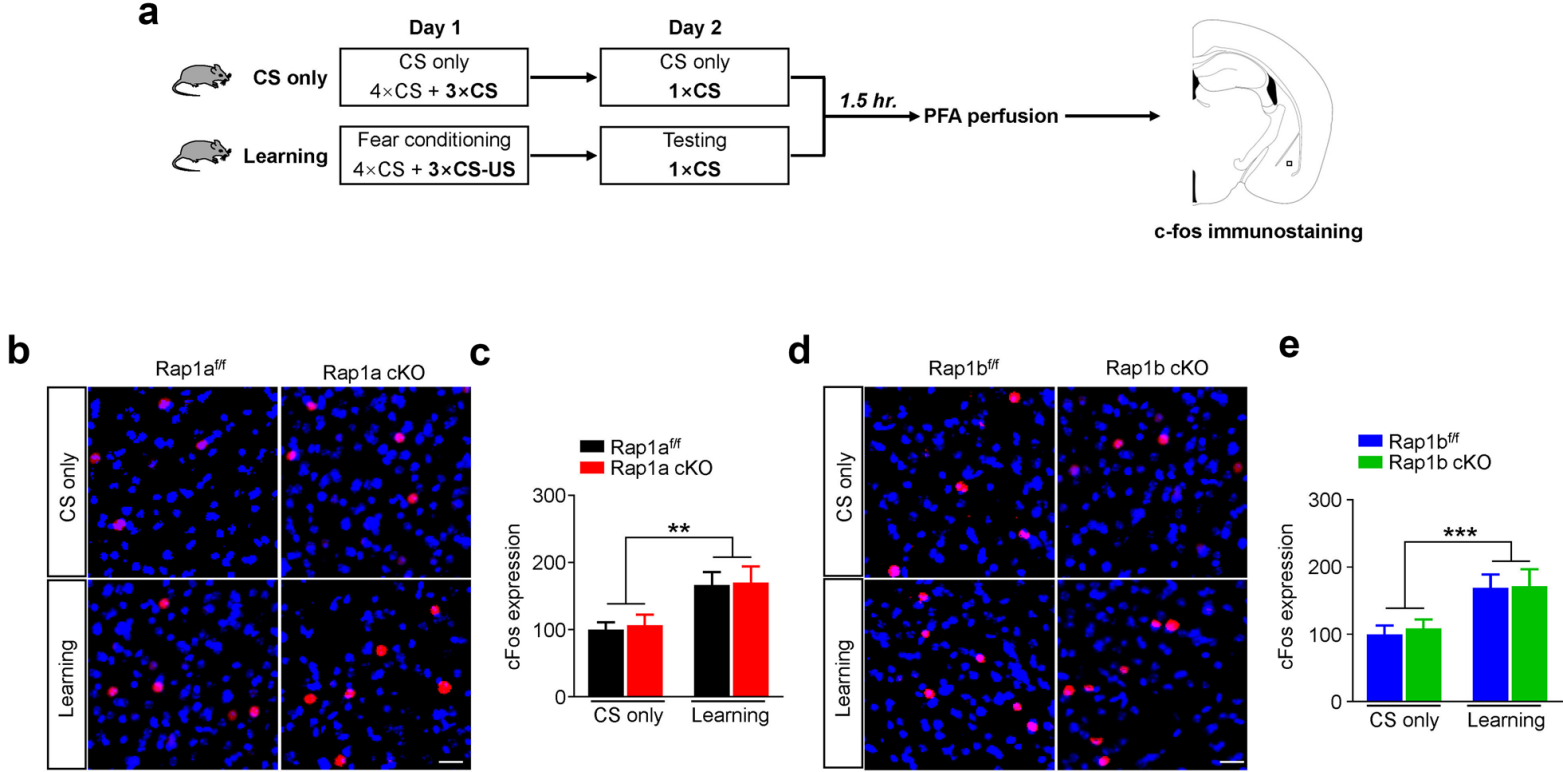
Rap1 is not required for retrieval-induced c-Fos expression in basolateral amygdala. **a** A schematic of the experimental configuration. **b** Representative images showing c-Fos expression in basolateral amygdala of Rap1a^f/f^ and Rap1a cKO mice. Slices were stained with anti-c-Fos (red) antibody, nuclei were showed by DAPI (blue). Scale bar, 30 μm. **c** Quantitative analysis of data in **b**. c-Fos expressions were normalized to Rap1a^f/f^ CS only group. For CS only group: n = 12 slices from 4 mice per group. For learning group: n = 16 slices from 4 mice per group. **d** Representative images showing c-Fos expression in basolateral amygdala of Rap1b^f/f^ and Rap1b cKO mice. Scale bar, 30 μm. **e** Quantitative analysis of data in **d**. c-Fos expressions were normalized to Rap1b^f/f^ CS only group. For CS only group: n = 13 slices from 4 mice per group. For learning group: n = 11 slices from 4 mice per group. Data are presented as mean ± SEM; **p < 0.01, ***p < 0.001

The amygdala and mPFC have been indicated as the kernel regions that mediates fear expression [17–21]. We thus determined whether retrieval-induced neuronal activation in these regions were altered by cKO of Rap1a and Rap1b. Neuronal activation was indicated by expression of immediate early gene, c-Fos (Fig. 3a). As shown in Fig. 3b–e, *Two-way* ANOVA revealed training but not genotype had significant main effect on the c-Fos expression in basolateral amygdala (BLA) of the two control-KO pairs mice (Rap1a; training: *F*_(1, 52)_ = 10.58, *p* = 0.002; genotype: *F*_(1, 52)_ = 0.067, *p* = 0.797; interaction: *F*_(1, 52)_ = 0.004, *p* = 0.952; Rap1b; training: *F*_(1, 44)_ = 13.28, *p* = 0.001; genotype *F*_(1, 44)_ = 0.095, *p* = 0.760; interaction: *F*_(1, 44)_ = 0.035, *p* = 0.852), indicating that Rap1 is not required for the altered neuronal activation by fear learning in basolateral amygdala.

**Fig. 4:**
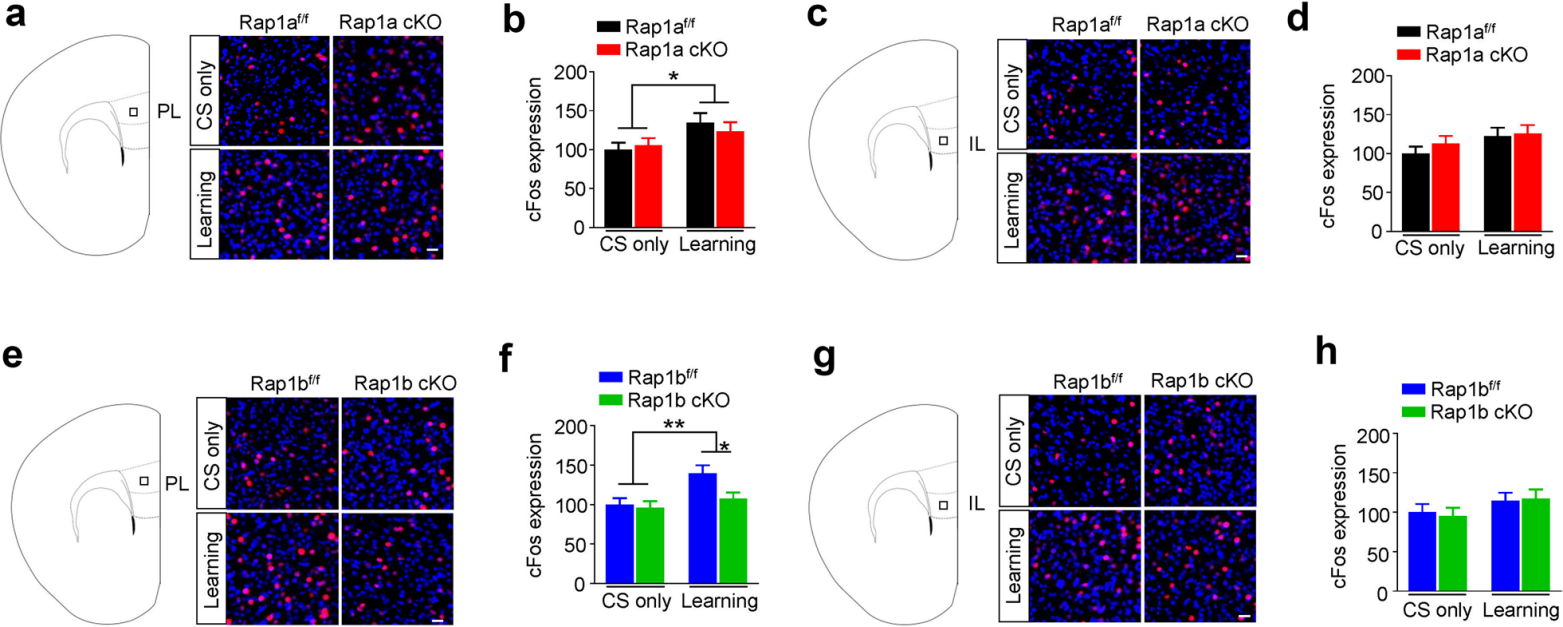
Rap1b is required for retrieval-induced c-Fos expression in PL. **a** Representative images showing c-Fos expression in PL of Rap1a^f/f^ and Rap1a cKO mice. Slices were stained with anti-c-Fos (red) antibody, nuclei were showed by DAPI (blue). Scale bar, 30 μm. **b** Quantitative analysis of data in **a**. c-Fos expressions were normalized to Rap1a^f/f^ CS only group. For CS only group: n = 12 slices from 4 mice per group. For learning group: n = 12 slices from 4 mice per group. **c** Representative images showing c-Fos expression in IL of Rap1a^f/f^ and Rap1a cKO mice. Scale bar, 30 μm. **d** Quantitative analysis of data in **c**. c-Fos expressions were normalized to Rap1a^f/f^ CS only group. For CS only group: n = 12 slices from 4 mice per group. For learning group: n = 12 slices from 4 mice per group. **e** Representative images showing c-Fos expression in PL of Rap1b^f/f^ and Rap1b cKO mice. Scale bar, 30 μm. **f** Quantitative analysis of data in **e**. c-Fos expressions were normalized to Rap1b^f/f^ CS only group. For CS only group: n = 12 slices from 4 mice per group. For learning group: n = 13 slices from 4 mice per group. **g** Representative images showing c-Fos expression in IL of Rap1b^f/f^ and Rap1b cKO mice. Scale bar, 30 μm. **h** Quantitative analysis of data in **g**. c-Fos expressions were normalized to Rap1b^f/f^ CS only group. For CS only group: n = 12 slices from 4 mice per group. For learning group: n = 13 slices from 4 mice per group. Data are presented as mean ± SEM; *p < 0.05, **p < 0.01

Next, we investigated whether Rap1a or Rap1b cKO affect retrieval-induced neuronal activation in PL and IL subregions of mPFC. As shown in Fig. 4a–d, *Two-way* ANOVA revealed training had significant main effect on the c-Fos expression in PL of two control-KO pairs mice (Rap1a; *F*_(1, 44)_ = 6.184, *p* = 0.017; Rap1b; *F*_(1, 46)_ = 8.596, *p* = 0.005). In addition, Rap1b cKO but not Rap1a cKO had significant main effect of genotype on the c-Fos expression in PL (Rap1a; *F*_(1, 44)_ = 0.078, *p* = 0.782; Rap1b; *F*_(1, 46)_ = 4.160, *p* = 0.047). Remarkably, *Post-hoc* analysis revealed that Rap1b cKO decreased c-Fos expression after fear learning (Rap1b^f/f^ vs Rap1b cKO, *p* = 0.024), indicating that Rap1b but not Rap1a is required for the increased neuronal activation by fear learning in PL. Moreover, As shown in Fig. 4e–h, *Two-way* ANOVA revealed neither training nor genotype had significant main effect on c-Fos expression in IL of two control-KO pairs mice (Rap1a; training: *F*_(1, 44)_ = 2.971, *p* = 0.092; genotype: *F*_(1, 44)_ = 0.631, *p* = 0.431; interaction: *F*_(1, 44)_ = 0.228, *p* = 0.636; Rap1b; training: *F*_(1, 46)_ = 2.906, *p* = 0.095; genotype: *F*_(1, 46)_ = 0.134, *p* = 0.907; interaction: *F*_(1, 46)_ = 0.108, *p* = 0.6744), indicating that Rap1 is not required for the retrieval-induced neuronal activation in IL. Our findings are consistent with previous reports showing that reduced PL activity impairs the expression of learned fear [17]. Altogether, these findings suggest that Rap1b in PL plays a crucial role in regulating fear memory expression.

### Rap1a and Rap1b are dispensable for the innate fear to predator odor

**Fig. 5:**
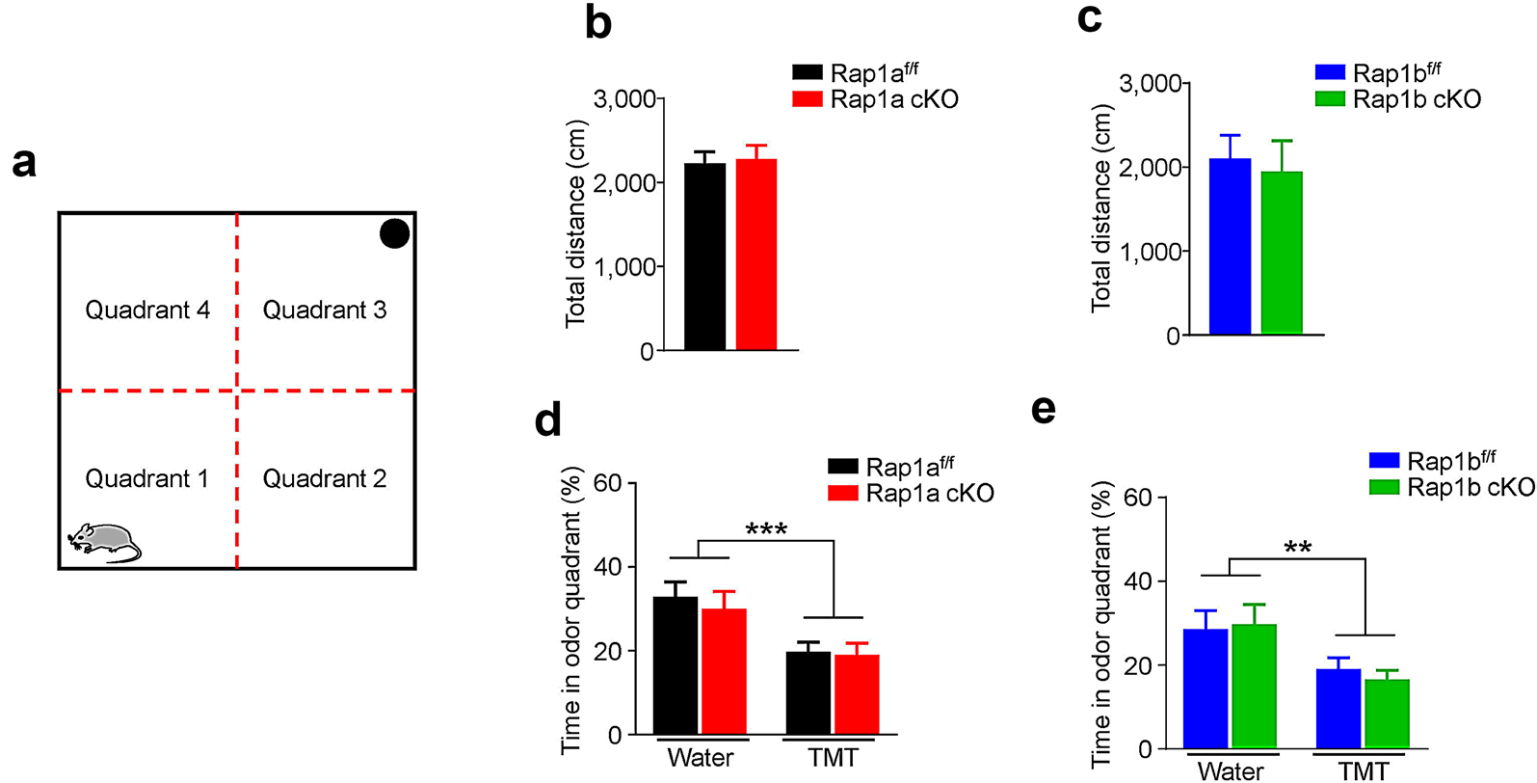
Rap1 is not required for TMT-evoked innate fear responses. **a** Images of the four-quadrant behavior chamber. The black circle indicates the position of water or TMT containing filter paper. **b** No difference in total distance traveled between Rap1a^f/f^ and Rap1a cKO mice. n = 10 mice per group. **c** No difference in total distance traveled between Rap1b^f/f^ and Rap1b cKO mice. n = 9 mice per group. **d** Average of duration spent in the cue quadrant of Rap1a^f/f^ and Rap1a cKO mice, in the present of water or TMT. n = 10 mice per group. **e** Average of duration spent in the cue quadrant of Rap1b^f/f^ and Rap1b cKO mice, in the present of water or TMT. n = 9 mice per group. Data are presented as mean ± SEM; **p < 0.01, ***p < 0.001

We next investigated the possible role of Rap1 in the innate fear of mice. To this end, the innate fear response was assessed in the four-quadrant chamber in response to TMT, a sulfur-containing compound isolated from the anal gland of fox that is widely used to evoke unconditioned fear in rodents (Fig. 5a) [22]. Both Rap1a and Rap1b cKO mice traveled a similar distance to their controls (Fig. 5b, c), indicating unaltered locomotor activity. For two control-KO pairs mice, the time they spent in the quadrant was decreased when TMT was present, compared with water (*Two-way* ANOVA; For Rap1a, odor: *F*_(1, 36)_ = 13.22, *p* = 0.001; For Rap1b, odor: *F*_(1, 32)_ = 9.145, *p* = 0.005; Fig. 5d, e), demonstrating presence of innate fear in all groups. Moreover, no effect of genotype was observed on the TMT-induced decrease of time spent in the odor quadrant (*Two-way* ANOVA; For Rap1a, *F*_(1, 36)_ = 0.307, *p* = 0.583; For Rap1b, *F*_(1, 32)_ = 0.030, *p* = 0.863; Fig. 5d, e), indicating neither Rap1a nor Rap1b cKO in forebrain affect the fear response to TMT.

## Discussion

Rap1a and Rap1b, two members of Rap superfamily, share highly homologous amino acid sequences (~ 85%) [15]. Previous studies investigating the functional and developmental role of Rap1 did not distinguish the functions between the two isoforms [9, 14, 23]. In the central nervous system, however, an increasing number of studies suggest that they may possess different functions. For example, a previous report showed that Rap1a, rather than Rap1b, regulates cell migration of developing neocortex [13]; while Rap1b makes greater contributions to axons development [24, 25]. Moreover, a recent study suggested a mediating effect of synaptic Rap1b in chronic cocaine treatment-induced bidirectional synaptic remodeling of medium spiny neurons in the nucleus accumbens [26]. Our previous work has demonstrated that conditional knockout of Rap1 in the forebrain impairs fear learning [16], whereas the exact role of the two isoforms of Rap1 in fear learning remains unclear. Here, we found that ablating Rap1b, but not Rap1a, could effectively replicate the fear learning deficits of rap1a/rap1b double KO mice, indicating an essential role of Rap1b in fear expression. Our data thus provide new evidence for the differentiated function between the two Rap1 isoforms.

Amygdala and mPFC are critical brain areas engaged in fear learning and memory, and the discrepancies of neural activity of these two regions always underlie the fear level bias [17–20]. In human, fMRI results suggest a positive correlation between the amygdala and dorsal mPFC activity and skin conductance responses [27, 28]. By contrast, a negative correlation exists between ventral mPFC neural activity and heart rate [29]. In rodents, large amounts of evidence indicate a higher level of fear expression coincides with increased PL and/or BLA neural activity [30, 31], and augmented IL activity is linked to constrained fear [32–34]. As such, abnormal neural activity in the above mentioned three brain areas can readily cause irregular fear expression. In the current study, the results of c-Fos immunostaining, which is another common approach to assess neuronal activity, showed suppression PL neuronal activity in Rap1b cKO mice following the retrieval of fear memory, a possible mechanism accounting for the fear learning deficits in Rap1 cKO mice.

In sharp contrast to acquired fear, mutation of either Rap1a or Rap1b had little effect on TMT-induced expression of innate fear. The defensive behavior elicited by TMT is independent of the experience of danger and threat [35]. In contrast, the expression of learned fear is a process of extracting the associative memory of conditioned and unconditioned stimuli [36]. Rap1 has been documented enormously in regulating synaptic plasticity which contributes to various types of learning and memory including fear learning [16, 23, 37, 38]. However, to date, there’s no evidence indicating neuronal plasticity underlies innate fear, yet [35], this could be one reason for the failure in observing changes in innate fear after Rap1(a/b) deletion in forebrain pyramidal neurons. Another scenario is plausible that TMT exposure is a strong stress paradigm that causes ceiling effect on the adaptive behavior, alteration of which is tough [39].

## Conclusions

Our study demonstrated that the genetic deletion of Rap1b, but not Rap1a, could effectively impair fear memory retrieval, whilst neither Rap1 isoform affected the habituation or fear acquisition. Furthermore, knockout of Rap1a or Rap1b had no effect on TMT-induced expression of innate fear. In addition, immunostaining studies indicate a differential activation of PL neurons in Rap1b rather than Rap1a cKO mice, which may contribute to the discrepant fear expression between the Rap1b^f/f^ and Rap1b cKO mice.

## Materials and methods

### Animals

*CaMKIIα-Cre, Rap1a-floxed (Rap1a*^*f/f*^*) and Rap1b-floxed (Rap1b*^*f/f*^*)* mice were described previously [16, 40]. Mice were housed under 12-h light/dark cycle and 22 ± 2 °C. All experiment procedures were approved by the Institutional Animal Care and Use Committee of Nanchang University.

### Quantitative real-time PCR

Total RNA was isolated from mouse cerebral cortex using TRIzol reagent (Invitrogen). cDNA was synthesized using a cDNA synthesis kit (GoScript™ Reverse Transcription System, Promega). The real-time PCR was performed with SYBR Green qPCR mastermix (Qiagen) on StepOnePlus real-time PCR system (Applied Biosystems). Glyceraldehyde 3-phosphate dehydrogenase (Gapdh) was used as internal control. The primers used were as follows: *rap1a* (F:5′-GCATCATGCGTGAGTACAAG’; R:5′- ACCTCGACTTGCTTTCTGTAG-3′); *rap1b* (F:

5′-GTGAATATAAGCTCGTCGTGC-3′; R: 5′-ACACTGCTGTGCATCTACTTC-3′); *gapdh* (F: 5′-AAGGTCATCCCAGAGCTGAA-3′; R: 5′-CTGCTTCACCACCTTCTTGA-3′).

### Behavioral analysis

Adult male mice (8–10 weeks) were used in behavioral tests. Mice were handled by investigators for 3 days before auditory fear conditioning and innate fear test. Auditory fear conditioning was performed as previously described with minor modification [16]. After adaptation for 2 days (5 min per day) in context A (white light, fans, metal grid floor and 4% acetic acid), mice were subjected to four presentations of the tone (conditioned stimulus, CS) (12 kHz, 80 dB, 10 s) in the absence of foot shocks for habituation and were subsequently fear-conditioned with three presentations of the tone that co-terminated with a 0.5 mA foot shock lasting 1 s (unconditioned stimulus, US). The intertrial interval (ITI) between CS presentations was presented in variable intervals (30–90 s). After an additional 30 s in the chamber, mice were removed from the chamber and returned to their home cage. After 24 h, 30-s tone CS was presented in the absence of the US in context B (red light, covered metal grid floor and 75% ethanol). Percentage of freezing time was measured during presence of tone. Freezing was monitored and analyzed by Video Freeze® fear conditioning system (Med Associates Inc., USA).

Innate fear test was conducted in an open-field chamber (50 × 50 cm^2^) and this chamber is divided into four equal quadrants (quadrant 1–4). First, the water was placed in quadrant 3 and the tested mouse was placed in quadrant 1, the duration of stay in quadrant 3 and total walking distance were monitored and measured for 10 min by an overhead tracking system (Med Associates Inc., USA). Subsequently, the water was replaced by 2,5-Dihydro-2,4,5-Trimethylthiazoline (TMT), and mouse was placed in zone 1 again and the duration of stay in quadrant 3 was measured. Chamber was cleaned with 70% ethanol and opened for ventilation between mice. Mice were exposed to each tested odor (water or TMT, respectively) only once.

### c-Fos immunohistochemistry

Immunohistochemistry was performed as previously described [41]. Briefly, anesthetized mice were perfused transcardially with 4% paraformaldehyde (PFA) in phosphate-buffered saline (PBS), pH 7.4. After postfixation in 4% PFA overnight, brains were sectioned into 30-µm-thick slices with VT1000S vibratome (Leica Microsystems, Germany). Slices were blocked with PBS containing 0.1% Triton X-100 and 10% normal donkey serum for 2 h at room temperature (RT), followed by incubation of primary c-Fos antibody (1:500) overnight at 4 °C. After washing with PBS for 3 times, samples were incubated with secondary antibodies for 2 h at RT and washed with PBS for 3 times. Finally, slices were mounted onto the slides with Fluoromount™ Aqueous Mounting Medium (Sigma-Aldrich), and images were taken by FV1000 confocal microscope (Olympus Corp, Japan).

### Statistical analysis

Data were analyzed with GraphPad Prism software. For analysis between two groups, the two-tailed unpaired Student’s t-test was used. For analysis of two groups at multiple time points, two-way ANOVA was used, followed by Bonferroni post hoc multiple comparison test. Data were presented as mean ± SEM. *p*-value < 0.05 was considered statistically significant.

## Data Availability

All data generated or analyzed during this study are included in this published article.

## Abbreviations

KO: Knockout
PL: Prelimbic cortex
IL: Infralimbic cortex
TMT: 2,5-Dihydro-2,4,5-Trimethylthiazoline
cKO: Conditional knockout
BDNF: Brain-derived neurotrophic factor
mPFC: Medial prefrontal cortex
PCR: Polymerase chain reaction
CaMKIIα: Ca2 + /calmodulin-dependent protein kinase II α subunit
BLA: Basolateral amygdala
ITI: Intertrial interval
PFA: Paraformaldehyde
PBS: Phosphate-buffered saline
DAPI: 4′,6-Diamidino-2-phenylindole
